# Establishment and Histopathological Characterization of a KYSE-30 Subcutaneous Xenograft Model of Esophageal Squamous Cell Carcinoma

**DOI:** 10.3390/cancers18101540

**Published:** 2026-05-10

**Authors:** Pavel A. Solopov, Alayna Enos, Mantas Silkunas, Giedre Silkuniene, Eleni Zivla, Andrei G. Pakhomov, Olga N. Pakhomova

**Affiliations:** 1 Frank Reidy Research Center for Bioelectrics, Old Dominion University, Norfolk, VA 23529, USA; arobe056@odu.edu (A.E.); msilkuna@odu.edu (M.S.); gsilkuni@odu.edu (G.S.); ezivl001@odu.edu (E.Z.); apakhomo@odu.edu (A.G.P.); opakhomo@odu.edu (O.N.P.); 2 Department of Electrical and Computer Engineering, Old Dominion University, Norfolk, VA 23529, USA

**Keywords:** ESCC, xenograft model, KYSE-30, histopathology, esophageal cancer

## Abstract

**Background/Objectives:** Esophageal squamous cell carcinoma (ESCC) is an aggressive malignancy with poor prognosis and limited treatment options, highlighting the need for reliable preclinical models for translational research. Although xenograft models are widely used in cancer biology, systematic characterization of subcutaneous ESCC models under defined implantation conditions remains limited. The objective of this study was to systematically compare implantation conditions and define histopathological and growth-related parameters that enable reproducible tumor establishment while maintaining a practical experimental window for therapeutic studies. **Methods:** We established and characterized a subcutaneous ESCC xenograft model using human ESCC cell line KYSE-30 implanted into immunodeficient J:NU mice. Tumor growth dynamics were compared under three implantation conditions: cells suspended in phosphate-buffered saline (PBS), Matrigel (Corning, basement membrane matrix), or Matrigel diluted with PBS. **Results:** Tumors generated from Matrigel-embedded cells exhibited rapid growth, reaching substantial size within approximately one week after implantation, which limited their utility for controlled therapeutic studies. In contrast, tumors generated from cells suspended in Matrigel + PBS demonstrated slower but consistent expansion, providing a more suitable and reproducible experimental window for therapeutic intervention. Cells injected in PBS alone showed inefficient tumor establishment. Histopathologic and immunohistochemical analyses revealed matrix-dependent differences in necrosis, stromal organization, and proliferative activity, while confirming epithelial tumor identity and baseline vascularization. **Conclusions:** Collectively, these findings define a reproducible KYSE-30 subcutaneous xenograft model and identify Matrigel dilution with PBS as an optimal implantation strategy that balances reliable tumor engraftment with controlled tumor growth suitable for therapeutic studies.

## Simple Summary

Esophageal cancer is an aggressive disease with limited treatment options, highlighting the need for reliable experimental tumor models. In this study, we established a mouse model using human esophageal cancer cells and examined how different injection conditions affect tumor growth and tissue structure. A key focus of this work was detailed histological evaluation to assess tumor organization, cell proliferation, and areas of cell death. We found that the composition of the injection matrix strongly influences not only how fast tumors grow, but also their internal architecture and viability. In particular, a mixture of Matrigel and saline produced consistent tumor formation with balanced growth and more uniform tissue structure. These findings provide practical guidance for generating reproducible tumor models and emphasize the importance of histological assessment in improving the reliability of preclinical cancer research.

## 1. Introduction

Esophageal squamous cell carcinoma (ESCC) is the most common histologic subtype of esophageal cancer worldwide and remains a major contributor to global cancer mortality, particularly in high-incidence regions such as East Asia, Eastern Europe, and parts of Africa [1,2,3]. Despite advances in multimodal therapy, long-term survival remains poor due to aggressive tumor biology, late diagnosis, and early metastasis [1,4]. ESCC is strongly associated with environmental and lifestyle risk factors, particularly tobacco and alcohol use, which act synergistically to increase carcer risk [5,6], as well as dietary carcinogens, micronutrient deficiencies, and chronic mucosal injury, including repeated exposure to very hot beverages [1,7,8]. These factors promote cumulative genetic and epigenetic alterations in the esophageal squamous epithelium, that ultimately drive malignant transformation, invasive growth, and stromal remodeling [4,9].

Preclinical models are critical for understanding ESCC biology and testing emerging therapies. Xenograft models in immunodeficient mice offer practical and reproducible platform for studying tumor growth kinetics, stromal interactions, and therapeutic response in vivo [10,11]. Subcutaneous xenografts are particularly attractive because they are technically simple, enable reliable tumor monitoring, and are well suited for longitudinal measurements, making them especially valuable for mechanistic and translational studies [11,12]. However, variations in tumor take rate, growth dynamics, vascularization, and extracellular matrix composition can influence experimental outcomes and therapeutic interpretation [13]. Comprehensive histopathological and microenvironmental characterization is therefore essential to ensure reproducibility, biological relevance, and suitability for downstream preclinical applications.

KYSE-30 is a well-established human ESCC cell line belonging to the KYSE series originally described by Shimada and colleagues and has been widely utilized in mechanistic and translational oncology research due to its robust growth characteristics and reproducible biological behavior [14]. The KYSE panel was developed to represent diverse clinicopathological and molecular features of primary ESCC, providing valuable experimental models for studying tumor biology and therapeutic response. When implanted subcutaneously into immunodeficient mice, KYSE-30 cells readily form xenograft tumors that can be monitored longitudinally to evaluate tumor growth dynamics and therapeutic response [12]. However, detailed and systematic characterization of xenograft histopathology, stromal composition, vascularization, proliferative activity, and baseline apoptotic index remains critical to ensure reproducibility and accurate interpretation of therapeutic outcomes across laboratories and experimental paradigms [10,11]. Such comprehensive profiling is particularly important in rapidly growing ESCC xenograft models, where extracellular matrix composition and tumor vascular architecture can influence tumor growth kinetics, drug delivery, and treatment response. Despite widespread use of KYSE-30 xenograft models, systematic comparisons of implantation conditions and their impact on tumor growth kinetics and baseline tumor microenvironment remain limited. In particular, how extracellular matrix composition influences tumor establishment, necrosis, stromal organization, and proliferative activity in this model has not been well defined.

In the present study, we directly compare three commonly used implantation strategies (phosphate-buffered saline (PBS), Matrigel, and Matrigel diluted with PBS) and provide integrated characterization of tumor growth dynamics and baseline histopathologic features. Matrigel (Corning) is a basement membrane extract rich in laminin, collagen IV, and growth factors that support tumor cell survival and engraftment. This approach enables identification of implantation conditions that ensure reliable tumor engraftment while maintaining a practical and reproducible window for therapeutic intervention. The tumor microenvironment plays a central role in regulating cancer progression, therapeutic responsiveness, and treatment resistance. Stromal collagen deposition and extracellular matrix remodeling alter tissue stiffness and interstitial pressure, thereby influencing tumor growth, drug penetration, and, in the context of bioelectrical therapies, electric field distribution within tumor tissue [15,16]. Increased extracellular matrix density has been shown to impair intratumoral transport and reduce therapeutic efficacy by limiting diffusion and vascular perfusion [16,17]. Tumor vascular density and architecture further modulate oxygenation status, nutrient delivery, and drug accessibility, while also contributing to heterogeneity in treatment response [18,19]. In parallel, proliferation and apoptosis indices provide critical insight into baseline tumor aggressiveness, cellular turnover, and potential sensitivity to cytotoxic interventions [20]. Comprehensive histopathologic and immunohistochemical profiling of xenograft tumors therefore enhances biological interpretation and strengthens the translational relevance of preclinical models.

The objective of this study was to compare commonly used implantation conditions and determine their impact on tumor growth dynamics and baseline histopathological features in a KYSE-30 ESCC xenograft model. We establish and characterize a subcutaneous ESCC xenograft model using the KYSE-30 cell line implanted into immunodeficient J:NU mice. This work provides a reproducible baseline platform for future mechanistic and therapeutic studies in ESCC.

## 2. Materials and Methods

### 2.1. Cell Culture and Preparation for Implantation

Human ESCC KYSE-30 cells (MilliporeSigma, Burlington, MA, USA) were cultured in a 1:1 mixture of RPMI-1640 and Ham’s F-12 medium with L-glutamine (Gibco, Thermo Fisher Scientific, Waltham, MA, USA) supplemented with 10% fetal bovine serum (Atlanta Biologicals, Norcross, GA, USA) and 100 U/mL penicillin–0.1 mg/mL streptomycin (Mediatech Cellgro, Herndon, VA, USA).

On the day of the experiment, cells were detached using TrypLE™ Express (Gibco, Thermo Fisher Scientific), counted, pelleted by centrifugation at 770× *g*, and washed twice with phosphate-buffered saline (PBS; Gibco, Thermo Fisher Scientific). Cells were then resuspended at 3 × 10^7^ cells/mL in PBS, Matrigel (Corning, Corning, NY, USA), or a 1:1 mixture of Matrigel and PBS, maintained on ice, and injected into animals within 1 h of preparation.

### 2.2. Animal Studies

All animal procedures were conducted in accordance with institutional guidelines and were approved by the Old Dominion University Institutional Animal Care and Use Committee (IACUC protocol #25-001). Immunodeficient J:NU male mice (Jackson Laboratory, Bar Harbor, ME, USA) aged 7–8 weeks, were housed under specific pathogen-free conditions with ad libitum access to food and water and maintained on a 12 h light/dark cycle. Animals were acclimated for at least 5 days prior to study initiation. A total of 45 mice were used in this study.

### 2.3. Study Design and Experimental Groups

KYSE-30 cells were prepared for implantation as described in the Cell Culturing Methods section. For tumor induction, mice were placed under biological hood, restrained manually and the left flank was disinfected with 70% ethanol. A suspension containing 1.5 × 10^6^ KYSE-30 cells in a total volume of 50 µL of PBS, Matrigel, or Matrigel + PBS was injected subcutaneously into the left flank using a 29G needle. The inoculum size (1.5 × 10^6^ cells per injection) was selected based on preliminary pilot experiments, in which lower cell numbers (1 × 10^6^) did not result in tumor establishment, and was further supported by commonly reported ranges in ESCC xenograft models [11,21,22]. Animals were immediately returned to their home cages after full ambulation.

**Figure 1:**
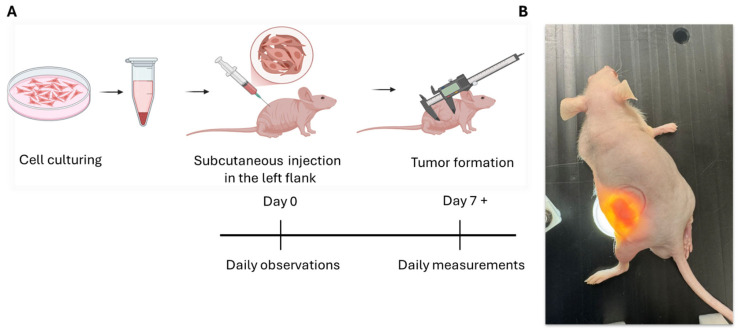
Overview of the KYSE-30 subcutaneous xenograft workflow in J:NU mice. (**A**) Schematic of study design: KYSE-30 cell preparation, subcutaneous implantation in the flank (day 0), tumor monitoring and caliper-based measurements. (**B**) Representative image of a J:NU mouse bearing a left flank subcutaneous tumor.

Mice were assigned to experimental groups based on the injection matrix/vehicle conditions: Group 1—(Cells in PBS): KYSE-30 cells suspended in 50 µL phosphate-buffered saline (PBS); Group 2 (Cells in Matrigel): KYSE-30 cells suspended in 50 µL of Matrigel; Group 3 (Cells in Matrigel + PBS): KYSE-30 cells suspended in 50 µL of Matrigel with PBS [1:1]; Group 4 (Matrigel Control): 50 µL of Matrigel. Each mouse received a single injection corresponding to one experimental condition. Mice were randomly assigned to groups at the time of injection and monitored daily for overall health and tumor-related endpoints (Figure 1).

### 2.4. Tumor Monitoring and Caliper Measurements

Tumor formation was assessed visually beginning on day 5 post-implantation and thereafter daily until study endpoint. Tumor size was measured using digital calipers, recording the longest (*a*) and shortest (*b*) perpendicular diameters. Tumor volume (mm^3^) was calculated as: *V* = *π*/6·*a*·*b*^·^^2^.

A target tumor volume range of 50–100 mm^3^ was defined as an optimal and reproducible window for potential therapeutic intervention, consistent with commonly used ranges in preclinical xenograft studies [23,24]. Mice were euthanized at or after day 8 post-implantation upon reaching predefined experimental endpoints or if humane endpoints were met, including ulceration, impaired ambulation, rapid weight loss (>15–20%), persistent lethargy, or tumor burden exceeding institutional limits (400 mm^3^). Animals exhibiting slower tumor growth were maintained beyond day 8 until endpoint criteria were achieved. Euthanasia was performed using isoflurane inhalation followed by cervical dislocation, in accordance with American Veterinary Medical Association.

### 2.5. Tissue Collection and Processing

At necropsy, tumors were excised and photographed. Tumors were then fixed in 10% neutral-buffered formalin for 72 h at room temperature, embedded in paraffin, sectioned at 5 µm, and mounted on glass slides for histological and immunohistochemical analyses. Serial sections from the same paraffin block were used for H&E, Masson’s trichrome, and immunostaining (Pan-CK, Ki-67, CD31, and TUNEL) as described below.

### 2.6. Histology-Anchored ROI Definitions

Necrotic regions were identified on H&E sections based on loss of nuclear staining and tissue architecture and quantified as a percentage of total tumor area when indicated. Masson’s trichrome staining was used for stromal assessment and collagen deposition. Pan-CK was used for epithelial identity confirmation, Ki-67 for proliferative activity, CD31 for assessment of microvascular density, and TUNEL assay for apoptotic index. Immunohistochemical quantifications were performed in viable tumor regions identified by morphology, excluding necrotic and acellular areas, using Fiji (ImageJ) software (v. 2.16.0). Ten microscopic fields per slide were analyzed, with n = 4 tumors per group.

## 3. Results

### 3.1. Tumor Growth Dynamics

**Figure 2:**
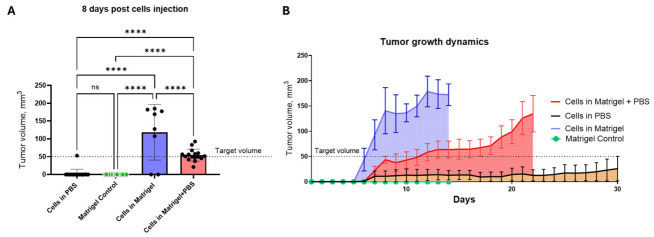
Tumor growth dynamics of KYSE-30 xenografts under different implantation conditions. (**A**) Tumor volume on day 8 post-implantation. (**B**) Longitudinal tumor growth curves for KYSE-30 cells implanted in Matrigel, Matrigel + PBS, or PBS alone. A Matrigel-only control group without tumor cells was included. Data are presented as mean ± SEM (n = 5–15 tumors per group). Statistical analysis was performed using one-way ANOVA with Kruskal–Wallis test. **** *p* < 0.00001, ns: not significant.

Tumor growth dynamics were monitored longitudinally following subcutaneous implantation of KYSE-30 cells under three experimental conditions (Figure 2). A control group received Matrigel without tumor cells to confirm the absence of spontaneous mass formation. As expected, no measurable tumor growth was observed in these animals throughout the observation period.

Tumors derived from KYSE-30 cells suspended in Matrigel exhibited the most rapid growth kinetics. Palpable tumors developed within the first week after implantation and increased rapidly in size, exceeding the target volume range for treatment initiation (50–100 mm^3^) by approximately days 7–10. In contrast, tumors generated from KYSE-30 cells suspended in a Matrigel + PBS mixture demonstrated slower initial growth but subsequently expanded in a more gradual and controlled manner, reaching measurable and experimentally manageable sizes (~400 mm^3^) between days 7 and 14. This growth pattern provided an optimal window for potential therapeutic intervention.

Cells injected in PBS without Matrigel showed minimal tumor establishment, with tumor diameters remaining near baseline levels throughout the monitoring period in most animals, indicating inefficient tumor engraftment in the absence of extracellular matrix support.

### 3.2. Tumor Necrosis Differs Across Matrix Conditions

**Figure 3:**
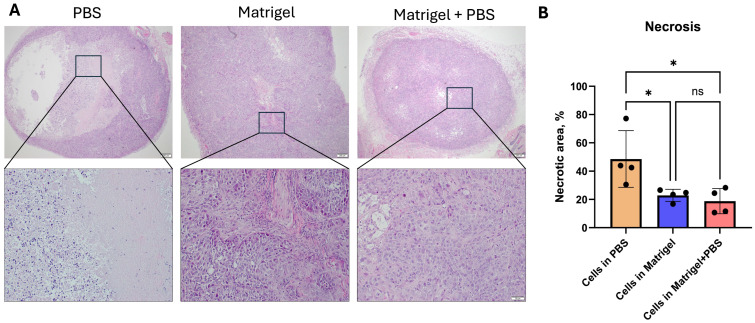
Histological assessment and quantification of tumor necrosis in KYSE-30 subcutaneous xenografts. (**A**) Representative hematoxylin and eosin (H&E)-stained sections of KYSE-30 tumors generated under different injection matrix conditions (PBS, Matrigel, and Matrigel + PBS). Upper panels show low-magnification views of entire tumor sections. Boxed regions indicate areas shown at higher magnification in the corresponding lower panels. Necrotic regions were defined as areas lacking nuclear staining and cellular architecture and were manually delineated based on H&E morphology prior to ImageJ quantification. (**B**) Quantification of necrotic fraction expressed as percentage of necrotic area relative to total tumor area. Data are presented as mean ± SEM (n = 4 tumors per group). Statistical analysis was performed using one-way ANOVA with Kruskal–Wallis test. * *p* < 0.05, ns: not significant. Scale bars: top row, 200 µm; bottom row, 50 µm.

Histological evaluation using H&E staining revealed distinct differences in tumor viability and necrotic architecture among experimental groups (Figure 3). Tumors generated from KYSE-30 cells injected in PBS exhibited prominent central necrosis characterized by extensive acellular eosinophilic regions lacking nuclear staining and preserved architecture. In contrast, tumors formed in the presence of Matrigel demonstrated reduced necrotic cores and a greater proportion of viable, densely cellular tumor tissue. Higher magnification analysis confirmed features of coagulative tumor necrosis in PBS tumors, including loss of nuclear detail, ghost cell outlines, and cellular debris at the interface between necrotic and viable regions. Matrigel-containing tumors displayed more preserved tumor nests with intact cellular morphology. Quantitative analysis of H&E sections demonstrated a significantly higher necrotic fraction in PBS-derived tumors compared with Matrigel-supported tumors (Figure 3B). Tumors established with Matrigel or Matrigel + PBS showed reduced necrotic area relative to total tumor area, indicating improved tumor viability and structural integrity.

### 3.3. Matrix Conditions Influence Stromal Architecture and Collagen Deposition

**Figure 4:**
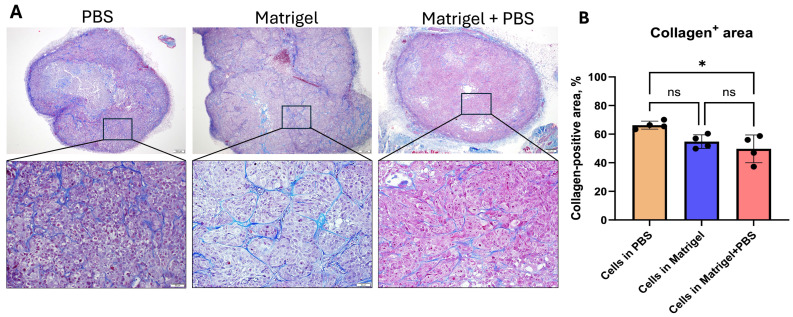
Stromal remodeling and collagen deposition in KYSE-30 subcutaneous xenografts. (**A**) Representative Masson’s trichrome-stained sections of KYSE-30 tumors generated under PBS, Matrigel, and Matrigel + PBS conditions. Upper panels show low-magnification views of entire tumor sections. Boxed regions indicate areas shown at higher magnification in the corresponding lower panels. Collagen fibers are stained blue, while tumor cells appear red/pink. (**B**) Quantification of collagen deposition expressed as percentage of collagen-positive area relative to total tumor area. Collagen-positive areas were quantified using color thresholding in Fiji (ImageJ) after standardization of hue, saturation, and brightness parameters across all samples. Data are presented as mean ± SEM (n = 4 tumors per group). Statistical analysis was performed using one-way ANOVA with Kruskal–Wallis test. * *p* < 0.05, ns: not significant. Scale bars: top row, 200 µm; bottom row, 50 µm.

To assess tumor stromal organization and extracellular matrix composition, sections were stained using Masson’s trichrome (Figure 4). Collagen fibers were visualized as blue staining, while tumor cells were identified by pink/red cytoplasmic staining. Tumors established in PBS displayed relatively limited and less organized collagen deposition, primarily localized at the tumor periphery and surrounding necrotic regions. In contrast, tumors formed in the presence of Matrigel demonstrated more pronounced and structured stromal networks, with collagen fibers interspersed between tumor nests and distributed throughout the viable tumor tissue. The Matrigel + PBS group exhibited intermediate stromal architecture with evident collagen deposition and preserved tumor organization. Quantitative analysis of collagen-positive area confirmed differences in extracellular matrix content among groups (Figure 4B). Matrigel-containing tumors exhibited modulated collagen deposition relative to PBS controls, reflecting altered stromal remodeling associated with matrix-supported tumor growth.

### 3.4. Epithelial Identity Confirmed by Pan-Cytokeratin Immunostaining

**Figure 5:**
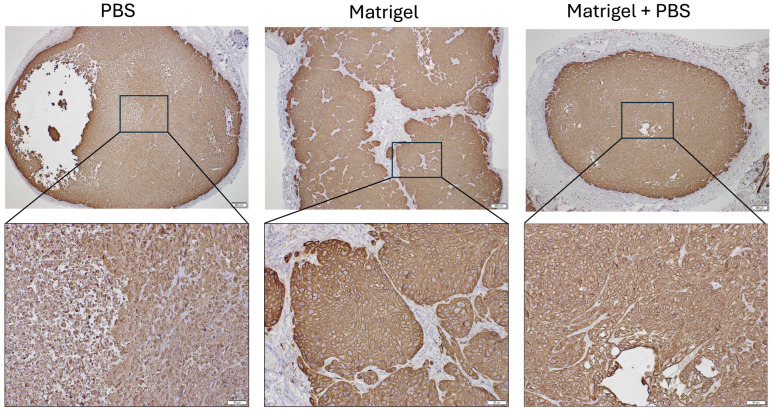
Pan-cytokeratin immunohistochemical staining confirms epithelial tumor identity in KYSE-30 xenografts. Representative Pan-CK-stained sections of KYSE-30 tumors generated under PBS, Matrigel, and Matrigel + PBS conditions. Upper panels show low-magnification views of whole tumor sections. Boxed regions indicate areas shown at higher magnification in the corresponding lower panels. Tumor cells demonstrate strong diffuse cytoplasmic staining, whereas surrounding stromal tissue remains largely negative. Scale bars: top row 200 µm; bottom row 50 µm. Representative images are shown from n = 4 tumors per group.

To confirm the epithelial origin of xenograft tumors, sections were stained for pan-cytokeratin (Pan-CK), a broad epithelial marker (Figure 5). All tumor groups demonstrated strong and diffuse cytoplasmic Pan-CK immunoreactivity within tumor cell nests, consistent with squamous epithelial differentiation. Stromal regions and surrounding host tissue were largely negative, confirming specificity of staining. Pan-CK expression was preserved across all matrix conditions, indicating stable epithelial phenotype and absence of overt phenotypic drift during tumor establishment. Necrotic regions and collagen-rich stromal compartments exhibited minimal Pan-CK immunoreactivity, consistent with the lack of viable epithelial tumor cells in these areas. No significant qualitative differences in Pan-CK expression were observed among groups.

### 3.5. Matrix-Dependent Differences in Tumor Vascularization

**Figure 6:**
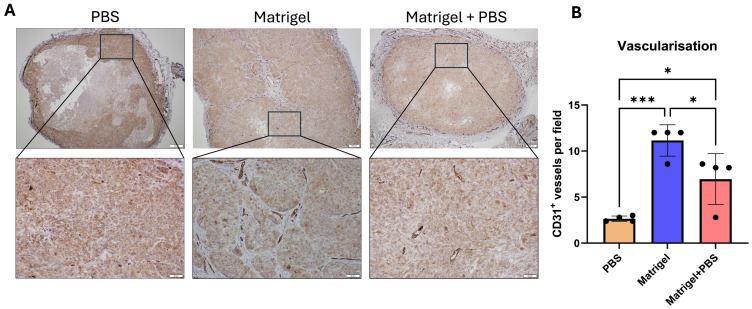
CD31 immunohistochemical visualization of tumor vasculature in KYSE-30 xenografts. (**A**) Representative CD31-stained sections of KYSE-30 tumors generated under PBS, Matrigel, and Matrigel + PBS conditions. Upper panels show low-magnification views of whole tumor sections. Boxed regions indicate areas shown at higher magnification in the corresponding lower panels. CD31-positive endothelial cells are visualized as brown-stained vascular structures within viable tumor tissue. (**B**) Quantification of CD31-positive vessels expressed as number of vessels per field. Vessel counts were performed in viable tumor regions, excluding necrotic and acellular areas. Ten representative fields per tumor were analyzed at consistent magnification; when fewer viable regions were available (PBS group), all available fields were included. Data are presented as mean ± SEM n = 4 tumors per group. Statistical significance was determined using one-way ANOVA with Kruskal–Wallis test; * *p* < 0.05, *** *p* < 0.001. Scale bars: top row 200 µm; bottom row 50 µm.

Tumor vascularization was evaluated by CD31 immunohistochemistry to assess microvessel density within viable tumor regions (Figure 6). CD31-positive endothelial structures were identified as discrete brown-stained, lumen-forming vascular profiles. Tumors established in PBS demonstrated relatively sparse and unevenly distributed microvessels, with vascular structures predominantly localized near the tumor periphery. In contrast, Matrigel-supported tumors exhibited more frequent and better-organized CD31-positive vessels distributed throughout viable tumor areas. The Matrigel + PBS group showed intermediate vascular architecture, with identifiable microvessels interspersed between tumor nests. Quantitative analysis of CD31-positive vessels confirmed these observations, with Matrigel tumors exhibiting the highest vessel density, followed by Matrigel + PBS, and PBS-derived tumors showing markedly reduced vascularization.

### 3.6. Matrigel Support Increases Tumor Proliferative Activity

**Figure 7:**
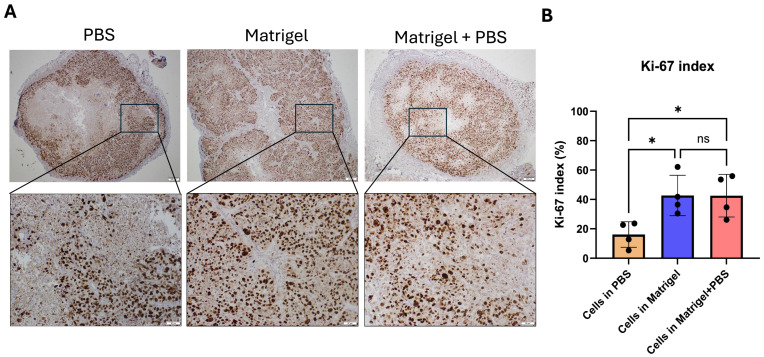
Ki-67 immunohistochemical analysis of proliferative activity in KYSE-30 xenografts. (**A**) Representative Ki-67-stained sections of KYSE-30 tumors generated under PBS, Matrigel, and Matrigel + PBS conditions. Upper panels show low-magnification views of whole tumor sections. Boxed regions indicate areas shown at higher magnification in the corresponding lower panels. Ki-67-positive nuclei are visualized as brown nuclear staining within viable tumor tissue. (**B**) Quantification of Ki-67 labeling index expressed as percentage of Ki-67-positive nuclei relative to total nuclei in viable tumor regions. Data are presented as mean ± SEM (n = 4 tumors per group) Statistical analysis was performed using one-way ANOVA with Kruskal–Wallis’s test, with significance indicated as * *p* < 0.05, ns: not significant. Scale bars: top row 200 µm; bottom row 50 µm.

To assess tumor cell proliferation, sections were stained for Ki-67, a nuclear marker of actively cycling cells (Figure 7). Ki-67-positive nuclei were identified as distinct brown nuclear staining within viable tumor regions. Tumors established in PBS demonstrated relatively lower Ki-67 labeling, with proliferative cells primarily localized at the tumor periphery. In contrast, tumors generated in the presence of Matrigel exhibited markedly increased Ki-67 positivity distributed throughout viable tumor compartments. The Matrigel + PBS group similarly demonstrated elevated proliferative activity compared to PBS controls. Quantitative analysis confirmed a significant increase in the Ki-67 labeling index in Matrigel-supported tumors relative to PBS-derived tumors (Figure 7B), indicating enhanced tumor cell proliferation under matrix-supported conditions.

### 3.7. Baseline Apoptotic Activity Assessed by TUNEL Staining

**Figure 8:**
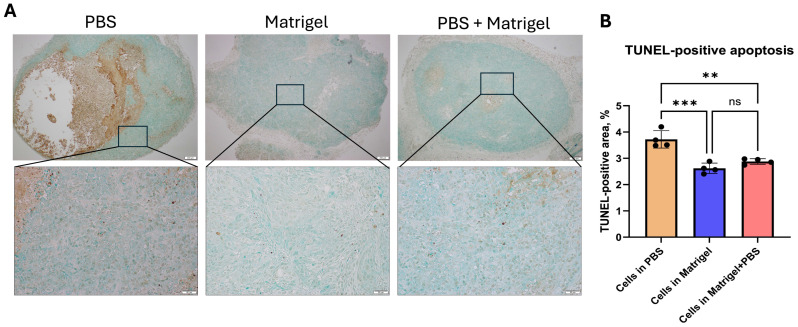
TUNEL analysis of baseline apoptotic activity in KYSE-30 xenografts. (**A**) Representative TUNEL-stained sections of KYSE-30 tumors generated under PBS, Matrigel, and Matrigel + PBS conditions. Upper panels show low-magnification views of whole tumor sections. Boxed regions indicate areas shown at higher magnification in the corresponding lower panels. TUNEL-positive nuclei are visualized as brown nuclear staining within viable tumor tissue. (**B**) Quantification of TUNEL labeling index expressed as percentage of TUNEL-positive nuclei relative to total nuclei in viable tumor regions. Data are presented as mean ± SEM (n = 4 tumors per group). Statistical analysis was performed using [one-way ANOVA with Kruskal–Wallis’s test. ** *p* < 0.01, *** *p* < 0.001, ns: not significant. Scale bars: top row 200 µm; bottom row 50 µm.

Apoptosis within xenograft tumors was evaluated using the TUNEL assay (Figure 8). TUNEL-positive nuclei were identified as brown-stained nuclear signals within viable tumor regions. PBS-derived tumors demonstrated increased TUNEL positivity, particularly at the interface between viable tumor tissue and necrotic areas. In contrast, tumors established in the presence of Matrigel exhibited fewer scattered TUNEL-positive nuclei within viable compartments. The Matrigel + PBS group showed a comparable pattern of apoptotic distribution. Quantitative analysis of TUNEL labeling index demonstrated modest differences in baseline apoptotic activity among groups (Figure 8, right panel), with PBS tumors exhibiting relatively higher apoptotic indices compared to matrix-supported tumors.

## 4. Discussion

The present study provides a systematic histopathological and microenvironmental characterization of a subcutaneous KYSE-30 xenograft model, highlighting how injection matrix conditions influence tumor architecture, viability, and proliferative dynamics. While subcutaneous xenografts are widely used in preclinical oncology, variability in stromal support and microenvironmental organization remains an underappreciated source of experimental heterogeneity. Our data demonstrate that matrix-supported implantation reduces necrotic burden and enhances proliferative activity compared with PBS-derived tumors, underscoring the biological consequences of tumor–stroma interactions during early tumor establishment.

Tumor growth kinetics in this study highlight the strong influence of implantation matrix composition on xenograft establishment and expansion. KYSE-30 cells suspended in Matrigel alone produced the most rapid tumor growth, with tumors exceeding the target volume range for treatment initiation within approximately one week after implantation. This accelerated growth resulted in a limited and less controllable intervention window. These findings are consistent with the established use of basement membrane extract (Matrigel) to enhance in vivo tumor initiation and expansion by providing extracellular matrix support that improves early cell survival, engraftment, and proliferation [25]. In contrast, partial dilution of Matrigel with PBS generated slower but reliable tumor expansion, producing a more practical treatment window of approximately 7–14 days. From an experimental standpoint, that growth profile is preferable because it preserves reproducible engraftment while avoiding excessively rapid overgrowth. Cells injected in PBS alone showed poor tumor establishment, which is also in line with standard xenograft methodology emphasizing the importance of matrix support for efficient implantation of cultured tumor cells in immunodeficient mice [26]. Collectively, these findings indicate that Matrigel + PBS provides the most useful balance between successful engraftment and controlled tumor growth for therapeutic studies in this KYSE-30 subcutaneous model.

A prominent finding was the markedly increased central necrosis in PBS-derived tumors. Large avascular necrotic cores are commonly observed in rapidly expanding solid tumors and are typically associated with inadequate vascular supply and diffusion-limited hypoxia [1,2]. Hypoxic stress contributes to tumor cell death in poorly perfused regions while simultaneously shaping tumor evolution and aggressiveness [3]. The reduced necrotic fraction observed in Matrigel-containing tumors suggests that early extracellular matrix support may facilitate improved structural integrity and possibly more efficient vascular integration, limiting ischemic collapse. Although CD31 staining in our study was qualitative, vascular structures were readily identifiable in all groups, indicating successful angiogenic recruitment consistent with prior reports that xenograft tumors rapidly establish host-derived vasculature [4,5].

Stromal remodeling, as visualized by Masson’s trichrome, revealed differences in collagen organization between matrix conditions. The extracellular matrix is increasingly recognized as a dynamic regulator of tumor growth rather than a passive scaffold [6]. Collagen architecture influences tumor stiffness, mechanotransduction signaling, and interstitial fluid pressure, all of which can affect proliferation and therapeutic penetration [7,8]. Matrix-supported tumors in our study displayed more organized stromal compartments interspersed among tumor nests, suggesting that initial implantation conditions can shape subsequent tumor microenvironment structure. These findings align with emerging evidence that matrix composition and mechanical context significantly modulate tumor cell behavior in vivo [9].

Consistent with reduced necrosis, Matrigel-supported tumors exhibited significantly increased Ki-67 labeling indices, indicating enhanced proliferative activity. Ki-67 is a well-established marker of cell-cycle progression and tumor growth fraction, frequently associated with aggressive biological behavior in solid tumors [10]. The inverse relationship observed between necrotic fraction and proliferative index across groups supports the concept that improved microenvironmental support promotes sustained tumor expansion. Importantly, proliferation differences were not accompanied by dramatic shifts in baseline apoptosis as assessed by TUNEL staining, which demonstrated only modest variation among groups. This suggests that matrix-dependent differences in tumor volume are primarily driven by proliferative dynamics rather than large changes in programmed cell death. Consistent with these findings, CD31-based analysis demonstrated that extracellular matrix support not only enhances tumor growth but also promotes the establishment of more developed and organized tumor vasculature.

Pan-cytokeratin staining confirmed stable epithelial phenotype across all experimental conditions, excluding the possibility that matrix support induced phenotypic drift or loss of squamous differentiation. Preservation of tumor identity is critical for ensuring translational validity, particularly in models intended for mechanistic and therapeutic studies.

This study has several limitations. First, it was performed using a single ESCC cell line (KYSE-30), which may limit generalizability across tumor subtypes. Second, the use of a subcutaneous xenograft model does not fully recapitulate the native esophageal microenvironment, including tissue-specific stromal interactions and anatomical constraints; alternative models such as orthotopic or patient-derived xenografts may provide additional translational relevance. Third, the use of immunodeficient mice precludes evaluation of tumor–immune interactions. Differences in tumor growth kinetics between experimental groups may result in variability in tumor maturity (“tumor age”) at the time of analysis, which could influence histopathological features such as necrosis and proliferative activity. Although this reflects inherent differences in tumor development under each condition, it may introduce bias when comparing histological parameters across groups. The present study did not evaluate the interaction between tumor cell inoculum size and implantation matrix composition. As inoculum size is an important determinant of tumor growth kinetics and microenvironmental features, its potential interaction with matrix conditions may influence the identification of optimal xenograft parameters. In addition, systemic effects were not evaluated in this study, as the primary focus was on local tumor characterization; therefore, potential systemic responses and off-target effects remain to be addressed in future studies. These limitations should be considered when interpreting the findings.

## 5. Conclusions

In summary, this study demonstrates that implantation matrix composition is an important determinant of tumor growth kinetics and histopathological features in KYSE-30 ESCC xenografts. While Matrigel alone promotes rapid tumor expansion that may limit experimental control, and PBS alone results in inefficient tumor establishment and increased necrosis, Matrigel diluted with PBS provides a balanced condition that supports reliable engraftment with controlled growth kinetics.

Histopathological analysis further indicates that matrix-supported tumors exhibit reduced necrotic burden, increased proliferative activity, and more organized stromal architecture compared to PBS-derived tumors.

These findings suggest that injection matrix conditions shape key microenvironmental parameters, including necrosis, stromal organization, and proliferative index, which are directly relevant for preclinical interpretation within the context of this model. As tumor oxygenation, extracellular matrix composition, and cellular turnover may influence drug penetration, radiotherapy sensitivity, and bioelectrical treatment distribution [7,11], standardizing implantation conditions and documenting baseline histopathologic characteristics may help improve reproducibility and cross-study comparability in ESCC xenograft research.

## Data Availability

The data presented in this study are available from the corresponding author upon reasonable request. All relevant data supporting the findings of this study are included within the article.

## References

[1] Abnet C.C., Arnold M., Wei W.Q. (2018). Epidemiology of Esophageal Squamous Cell Carcinoma. Gastroenterology.

[2] Bray F., Laversanne M., Sung H., Ferlay J., Siegel R.L., Soerjomataram I., Jemal A. (2024). Global cancer statistics 2022: GLOBOCAN estimates of incidence and mortality worldwide for 36 cancers in 185 countries. CA Cancer J. Clin..

[3] Sung H., Ferlay J., Siegel R.L., Laversanne M., Soerjomataram I., Jemal A., Bray F. (2021). Global Cancer Statistics 2020: GLOBOCAN Estimates of Incidence and Mortality Worldwide for 36 Cancers in 185 Countries. CA Cancer J. Clin..

[4] Rustgi A.K., El-Serag H.B. (2014). Esophageal carcinoma. N. Engl. J. Med..

[5] Islami F., Fedirko V., Tramacere I., Bagnardi V., Jenab M., Scotti L., Rota M., Corrao G., Garavello W., Schüz J. (2011). Alcohol drinking and esophageal squamous cell carcinoma with focus on light-drinkers and never-smokers: A systematic review and meta-analysis. Int. J. Cancer.

[6] Prabhu A., Obi K.O., Rubenstein J.H. (2014). Rubenstein, The synergistic effects of alcohol and tobacco consumption on the risk of esophageal squamous cell carcinoma: A meta-analysis. Am. J. Gastroenterol..

[7] Islami F., Boffetta P., Ren J.S., Pedoeim L., Khatib D., Kamangar F. (2009). High-temperature beverages and foods and esophageal cancer risk--a systematic review. Int. J. Cancer.

[8] Tran G.D., Sun X.D., Abnet C.C., Fan J.H., Dawsey S.M., Dong Z.W., Mark S.D., Qiao Y.L., Taylor P.R. (2005). Prospective study of risk factors for esophageal and gastric cancers in the Linxian general population trial cohort in China. Int. J. Cancer.

[9] Cancer Genome Atlas Research Network, Analysis Working Group: Asan University, BC Cancer Agency, Brigham and Women’s Hospital, Broad Institute, Brown University, Case Western Reserve University, Dana-Farber Cancer Institute, Duke University, Greater Poland Cancer Centre (2017). Integrated genomic characterization of oesophageal carcinoma. Nature.

[10] Hidalgo M., Amant F., Biankin A.V., Budinská E., Byrne A.T., Caldas C., Clarke R.B., de Jong S., Jonkers J., Mælandsmo G.M. (2014). Patient-Derived Xenograft Models: An Emerging Platform for Translational Cancer Research. Cancer Discov..

[11] Tentler J.J., Tan A.C., Weekes C.D., Jimeno A., Leong S., Pitts T.M., Arcaroli J.J., Messersmith W.A., Eckhardt S.G. (2012). Patient-derived tumour xenografts as models for oncology drug development. Nat. Rev. Clin. Oncol..

[12] Kerbel R.S. (2003). Human tumor xenografts as predictive preclinical models for anticancer drug activity in humans: Better than commonly perceived-but they can be improved. Cancer Biol. Ther..

[13] Morton J.J., Bird G., Refaeli Y., Jimeno A. (2016). Humanized Mouse Xenograft Models: Narrowing the Tumor-Microenvironment Gap. Cancer Res..

[14] Shimada Y., Imamura M., Wagata T., Yamaguchi N., Tobe T. (1992). Characterization of 21 newly established esophageal cancer cell lines. Cancer.

[15] Lu P., Weaver V.M., Werb Z. (2012). The extracellular matrix: A dynamic niche in cancer progression. J. Cell Biol..

[16] Stylianopoulos T., Jain R.K. (2013). Combining two strategies to improve perfusion and drug delivery in solid tumors. Proc. Natl. Acad. Sci. USA.

[17] Heldin C.H., Rubin K., Pietras K., Ostman A. (2004). High interstitial fluid pressure—An obstacle in cancer therapy. Nat. Rev. Cancer.

[18] Carmeliet P., Jain R.K. (2000). Angiogenesis in cancer and other diseases. Nature.

[19] Jain R.K. (2005). Normalization of tumor vasculature: An emerging concept in antiangiogenic therapy. Science.

[20] Scholzen T., Gerdes J. (2000). The Ki-67 protein: From the known and the unknown. J. Cell Physiol..

[21] Bibby M.C. (2004). Orthotopic models of cancer for preclinical drug evaluation: Advantages and disadvantages. Eur. J. Cancer.

[22] Fidler I.J. (1975). Biological behavior of malignant melanoma cells correlated to their survival in vivo. Cancer Res..

[23] Ho W.S., Wang H., Maggio D., Kovach J.S., Zhang Q., Song Q., Marincola F.M., Heiss J.D., Gilbert M.R., Lu R. (2018). Pharmacologic inhibition of protein phosphatase-2A achieves durable immune-mediated antitumor activity when combined with PD-1 blockade. Nat. Commun..

[24] Minnix M., Li L., Yazaki P.J., Miller A.D., Chea J., Poku E., Liu A., Wong J.Y.C., Rockne R.C., Colcher D. (2021). TAG-72-Targeted α-Radionuclide Therapy of Ovarian Cancer Using (225)Ac-Labeled DOTAylated-huCC49 Antibody. J. Nucl. Med..

[25] Benton G., Kleinman H.K., George J., Arnaoutova I. (2011). Multiple uses of basement membrane-like matrix (BME/Matrigel) in vitro and in vivo with cancer cells. Int. J. Cancer.

[26] Fridman R., Benton G., Aranoutova I., Kleinman H.K., Bonfil R.D. (2012). Increased initiation and growth of tumor cell lines, cancer stem cells and biopsy material in mice using basement membrane matrix protein (Cultrex or Matrigel) co-injection. Nat. Protoc..

